# Inexpensive Antimony Nanocrystals and Their Composites with Red Phosphorus as High-Performance Anode Materials for Na-ion Batteries

**DOI:** 10.1038/srep08418

**Published:** 2015-02-12

**Authors:** Marc Walter, Rolf Erni, Maksym V. Kovalenko

**Affiliations:** 1 Laboratory of Inorganic Chemistry, Department of Chemistry and Applied Biosciences, ETH Zürich, CH-8093 Zürich, Switzerland; 2 Laboratory for Thin Films and Photovoltaics, Empa – Swiss Federal Laboratories for Materials Science and Technology, CH-8600 Dübendorf, Switzerland; 3 Electron Microscopy Center, Empa – Swiss Federal Laboratories for Materials Science and Technology, CH-8600 Dübendorf, Switzerland

## Abstract

Sodium-ion batteries increasingly become of immense research interest as a potential inexpensive alternative to Lithium-ion batteries. Development of high-energy-density negative electrodes (anodes) remains to be a great challenge, especially because of significant differences between lithium and sodium chemistries. Two Na-ion anode materials – antimony (Sb) and phosphorus (P) – have been recently shown to offer excellent cycling stability (Sb) and highest known Na-ion charge storage capacity (P). In this work we report on the synergistic Na-ion storage in a P/Sb/Cu-nanocomposite, produced by mixing inexpensive colloidal Sb nanocrystals with red P and with copper (Cu) nanowires. In comparison to electrodes composed of only phosphorus, such P/Sb/Cu-composite shows much greater cycling stability providing a capacity of above 1100 mAh g^−1^ after 50 charge/discharge cycles at a current density of 125 mA g^−1^. Furthermore, P/Sb/Cu-composite also exhibits excellent rate-capability, with capacity of more than 900 mAh g^−1^ at a high charge/discharge current density of 2000 mA g^−1^.

Rechargeable batteries – which can store electricity of any origin in the form of chemical bond energy – are increasingly required to offer higher energy density, long cycle life and safety, all at sufficiently low cost of production. Sodium-ion batteries (SIBs) have long been neglected in favour of Lithium-ion batteries (LIBs), mainly due to the successful commercialization of LIBs early in 1990s. However, in light of the limited abundance and geographically uneven distribution of lithium salts (with main reserves in South America) concerns are raising regarding security of supply and cost of Li in the near future. Hence, for the growing need for batteries for electrical mobility and large-scale energy storage, conceptually identical SIBs are gaining attention as an economically viable alternative to LIBs^1^,^2^,^3^,^4^. While SIB cathodes have now reached similar performance to LIB cathodes^5^,^6^,^7^,^8^,^9^,^10^,^11^,^12^, the development of stable high-energy-density anode materials for SIBs still lacks well behind the Li-ion counterparts. In particular, silicon as well as graphite – which are two most successful LIB anode materials – have been reported to possess a negligible capacity for Na-ion storage^13^,^14^. Thus an extensive exploration of novel Na-ion anode materials has been launched in the recent years^15^,^16^,^17^,^18^,^19^,^20^,^21^,^22^,^23^,^24^,^25^,^26^,^27^,^28^,^29^,^30^,^31^,^32^.

Low cost and high Na-ion storage capacity are the major considerations for pre-selection of promising materials, whereas long cycle life has to be achieved *via* smart engineering of the electrodes at micro/nanoscale. In this regard, red phosphorus (P) – the most chemically and thermodynamically stable allotrope of P – is both cheap and offers the highest known theoretical capacities for Na-ion storage (2596 mAh g^−1^ for P
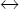
Na_3_P transition). However, achieving such high capacities with acceptable cycling stability is hampered by massive increase of volume upon sodiation (%Δ*V* = 291%)^21^ leading to the formation of cracks, loss of electrical contacts and fast capacity fading after several charge/discharge cycles. These difficulties are also commonplace for Si-based and other alloying anode materials in LIBs and are usually mitigated by nanostructuring of the active material^33^,^34^,^35^,^36^,^37^,^38^,^39^,^40^,^41^,^42^,^43^,^44^,^45^,^46^,^47^,^48^,^49^,^50^,^51^. Another common problem of P is the rather poor electronic conductivity and low diffusivity of Na^+^ ions. Red P is a rather soft material with a low melting point and can be processed into a micro/nanostructured battery anode by hand or ball-milling with amorphous carbon nanoparticles (NPs)^21^,^22^,^23^,^24^. Such composites do show record-breaking near-theoretical capacities, but suffer from capacity fading due to both inherent instability of P and far from optimal performance of present-day Na-ion electrolytes. The second most promising Na-ion anode material is Sb (and Sb-based compounds), with theoretical capacity of 660 mAh g^−1^ (for Sb
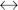
Na_3_Sb conversion) and good cycling stability^25^,^26^,^27^,^28^,^29^,^30^,^31^,^32^,^52^. We recently introduced monodisperse Sb nanocrystals (NCs), produced *via* organometallic synthesis in nonpolar organic solvents, showing excellent cycling stability and rate capability (retention of >80% of theoretical capacity at 20C-rate for charge and discharge, 1C-rate being current density of 660 mA g^−1^)^53^. Yet the fundamental drawback of organometallically prepared Sb NCs is the high cost of their synthesis and purification in organic solvents, using coordination compounds as precursors and expensive reducing agents.

In this study we thought to combine the best of P and Sb – much higher capacity of P and fast and stable cycling of Sb – into a functional synergy through the design of an inexpensive P-Sb composite. We used the insights gained from our recent study on monodisperse Sb NCs; in particular, the fact that the performance of Sb NCs is only moderately size-dependent as long as NCs are in 20–100 nm size range. Hence, for the present study we developed a facile low-cost synthesis of Sb NCs, which, despite broader size distribution, exhibit even better performance than organometallically-synthesized Sb NCs. We were interested to find whether good electrochemical properties of Sb-P composite can be achieved without nanostructuring by high-energy mechanical milling, but simply by one-pot mixing the commercial red P with Sb NCs, solvent, binder and conductive additive. Further, through the addition of copper nanowires (Cu NWs) we demonstrate the importance of tailoring the electronic conductivity and mechanic stability for enhancing electrochemical performance. The as-prepared P/Sb/Cu-composite delivers a capacity of >1300 mAh g^−1^ for 30 cycles corresponding to >80% of its theoretical capacity. Moreover, the P/Sb/Cu-composite shows outstanding rate capability retaining a capacity of >900 mAh g^−1^ at a current of 2000 mA g^−1^.

## Results

### Inexpensive synthesis and electrochemical performance of Sb NCs

**Figure 1:**
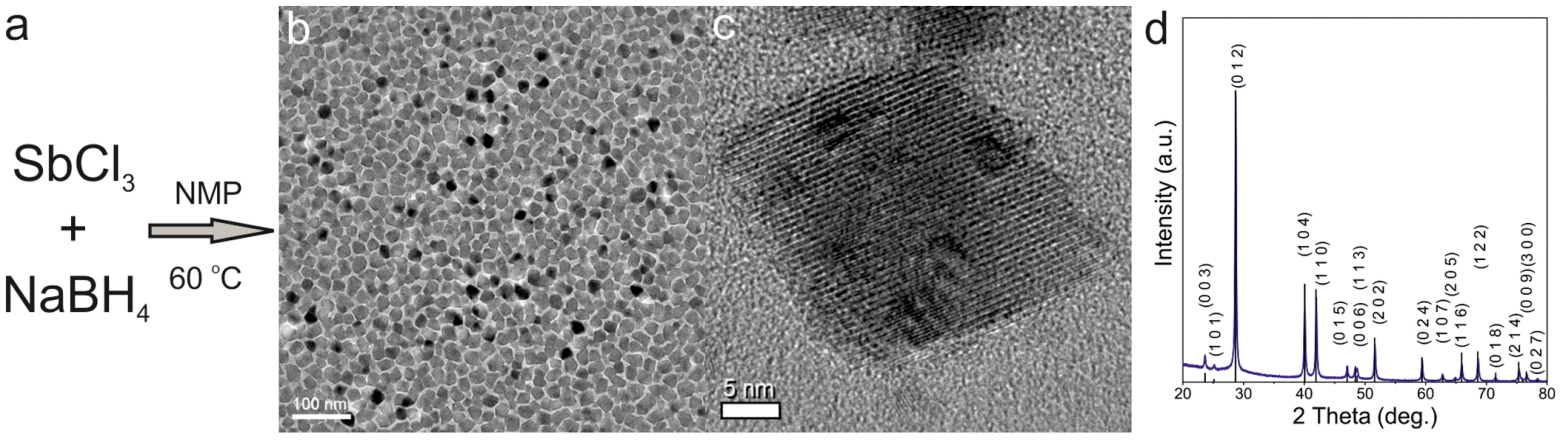
Synthesis and characterization of Sb NCs: (a) Reaction scheme; (b) TEM image; (c) high-resolution TEM image; (d) X-ray diffraction (XRD) pattern indexed to pure-phase hexagonal Sb (ICDD database, PDF Entry No.: 00-071-1173).

Colloidal NCs make for an ideal battery material: they can be safely and conveniently produced and handled as stable dispersions in common solvents. Using monodisperse Sb NCs, we recently reported that 20 nm NCs exhibit higher Li-ion and Na-ion storage capacities and improved cycling stability, especially at higher rates of charge and discharge, than both smaller ~10 nm particles and (sub)micron-sized Sb^53^. However, the synthesis of monodisperse Sb NCs involved expensive organic and coordination compounds (precursors, solvents, surfactants and reducing agents), as well as multiple steps of washing and removal of surface capping ligands. Yet a very important conclusion was obtained: mean particle size has a very modest effect on the electrochemical characteristics of Sb NCs, as long as it lies within the range of 20–100 nm, in striking contrast to, for instance, Li-ion storage in Sn NCs, showing satisfactory performance only at sizes of 10 nm and below^54^. Such intrinsic tolerance of Sb to crystallite size has prompted us to search for an inexpensive synthetic route, even if the “precision” of the synthesis is somewhat sacrificed. For this work, we developed a much simpler and cheaper (~1 USD/g) synthesis of Sb NCs *via* reduction of SbCl_3_ with NaBH_4_ at 60°C (for details, see the Experimental Section), yielding NCs of similar size as in a previous study, but at a cost that is lower by factor of ~100. The main features of this synthesis are the gram-scale and further up-scalable production with above 80% reaction yield, simple isolation of Sb NCs by centrifuging and convenient recycling of the solvent. The absence of surfactants obviates lengthy purification and surfactant-removal procedures. The only post-synthetic treatment is washing with water to remove the reaction byproducts (sodium chloride and borates). Figure 1 presents Sb NCs with the mean size of ~20 nm. The X-ray diffraction (XRD) pattern shows phase-pure, highly crystalline Sb NCs (space group N166, *R-3 m*, a = b = 0.4306 nm, c = 1.1288 nm; calculated crystallite size of 22 nm; see Supplementary Fig. S1 for Rietveld refinement), without any detectable traces of Sb oxides or reaction byproducts.

### Preparation and electrochemical performance of Sb-P nanocomposites

**Figure 2:**
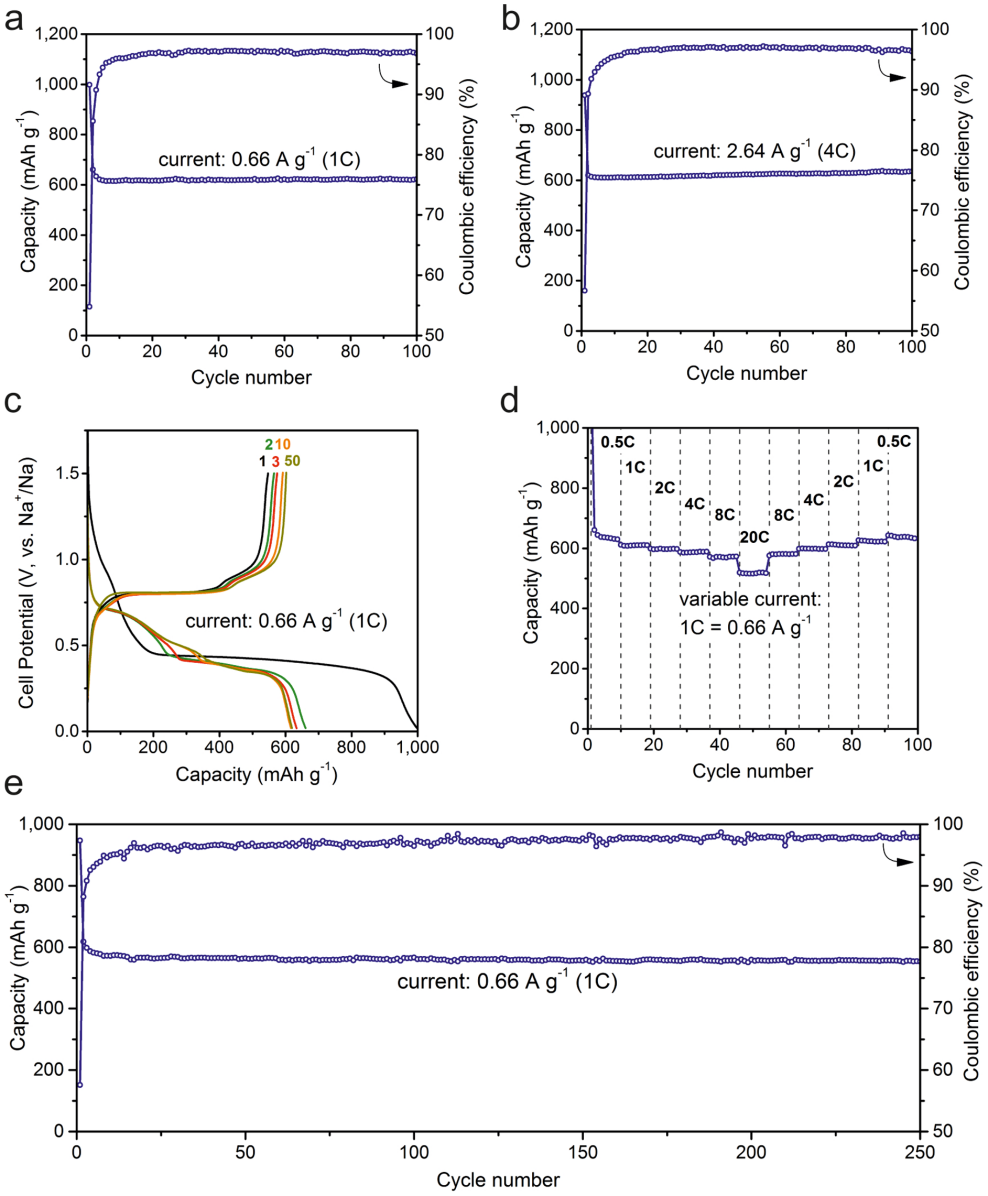
Electrochemical performance of ~20 nm Sb NCs in Na-ion coin-type half-cells: (a) and (b) illustrate capacity retention at a rate of 1C and 4C (1C = 0.66 Ag^−1^), respectively; (c) galvanostatic charge and discharge curves; (d) rate-capability tests (0.5–20C rate); (e) long-term cycling stability tests with enforced limitation of charge capacity to 550 mAh g^−1^. The composition of electrodes was Sb (64 wt%), CB (21 wt%) and CMC binder (15 wt%). 1 M NaClO_4_ in propylene carbonate with addition fluoroethylene carbonate (10 wt%, FEC) was used as electrolyte. All batteries were cycled in the 0.02–1.5 V potential range; capacities were normalized to the mass of active material.

Electrochemical performance of Sb NCs and of all other materials in this study has been tested in standard coin-type half-cells with metallic Na as counter electrode. Working electrodes were prepared by mixing Sb NCs (64 wt%), with carbon black (CB) as conductive additive (21 wt%) and carboxymethylcellulose (CMC, 15 wt%) as a water soluble polymeric binder, followed by doctor-blade casting of thus formed slurry on Cu foil (mass loading ~0.5 mg/cm^2^). 1 M NaClO_4_ in propylene carbonate was used as an electrolyte and fluoroethylenecarbonate (FEC) was used as electrolyte additive for stabilizing the solid-electrolyte interface (SEI)^46^. In spite of the broader size distribution compared to organometallically-synthesized Sb NCs, inexpensive Sb NCs developed in this study exhibit same or even better electrochemical characteristics (Fig. 2, for comparison see Supplementary Fig. S2).

Namely, near-theoretical capacity is obtained at 0.5–1C rates (1C corresponds to current density of 660 mA g^−1^) without noticeable deterioration in the first 100 cycles. The small increase of the charge capacity observed in the charge/discharge curves might be due to the fact that not all of the electrode material is fully active during the first cycles due to kinetic limitations. Considering the massive volume changes for the sodiation/desodation reaction most likely the electrodes undergo restructuring in the first cycles leading to slightly better ionic and electronic conductivity, which explains the small increase of the charge capacity. Rate-capability tests indicate retention of at least 85% of capacity at a high rate of 20C (13.2 A g^−1^) and full recovery of the capacity after decrease of the cycling rate to 0.5C. The rate-capability of Sb is unprecedented for Na-ion anodes, and is comparable to fastest Li-ion intercalation materials such as graphite^55^, and Li titanates^56^. In addition, at a relatively high current density of 660 mA g^−1^ our Sb NC anodes show excellent cycling stability for at least 250 cycles, when the charge capacity is limited to 550 mAh g^−1^ (Fig. 2e). Similarly, if the charge capacity is limited to 400 mAh g^−1^ Sb NCs retain their capacity for at least 400 cycles (Supplementary Fig. S3). Such impressively fast and stable operation of Sb anodes can be attributed to the synchronous effect of several properties. First, Sb is a good electronic conductor due to its semimetallic nature. Second, its crystalline structure is characterized by the low atomic packing factor of just 39%^57^ and the crystal structure is comprised of puckered layered planes with large channels for diffusion of alkali ions. Insertion of Na-ions into Sb involves only one crystalline (Na_3_Sb) and several amorphous phases, including amorphous Sb^52^. De-insertion primarily occurs as direct Na_3_Sb_hex_→Sb_amorphous_ transition. The reduced number of intermediate crystalline phases greatly enhances the conversion kinetics. The theoretical value for volumetric expansion upon full sodiation to hexagonal Na_3_Sb can be estimated from the difference in the molar volumes (%*V_m_*) between the final (*Na_3_Sb*) and the initial metallic (*Sb*) phases: %Δ*V* = 100% × [*V_m_*(*Na_x_Sb*)−*V_m_*(*Sb*)]/*V_m_*(*Sb*) = 290%. It is the effect of isotropic, nearly strain-free expansion/contraction of amorphous phases that can explain the tolerance of Sb to such drastic volumetric changes.

Fundamentally, the formation of composites opens up multiple new opportunities for tuning the electronic and ionic transport, surface chemistry, porosity and mean particle size, with strong implications for rate capability and cycling stability. The initial goal of this study was to obtain a compelling combination of the two materials: functional synergy of higher capacity of P with high-rate capability and high cyclability of Sb.

P undergoes very similar to Sb volumetric changes of ~290% upon full sodiation to Na_3_P^23^. At the same time, P shows much faster capacity fade^21^,^22^,^23^, which can be attributed to poor electronic and ionic conductivity. Replacing some of the P with Sb NCs might give rise to improved electrochemical properties as (sub)micron-sized P particles are more diluted in a conductive nanoscopic matrix of Sb and CB. For 1:1 mixture of Sb and P, one can expect capacities up to theoretical weighed average of 1628 mAh g^−1^.

**Figure 3:**
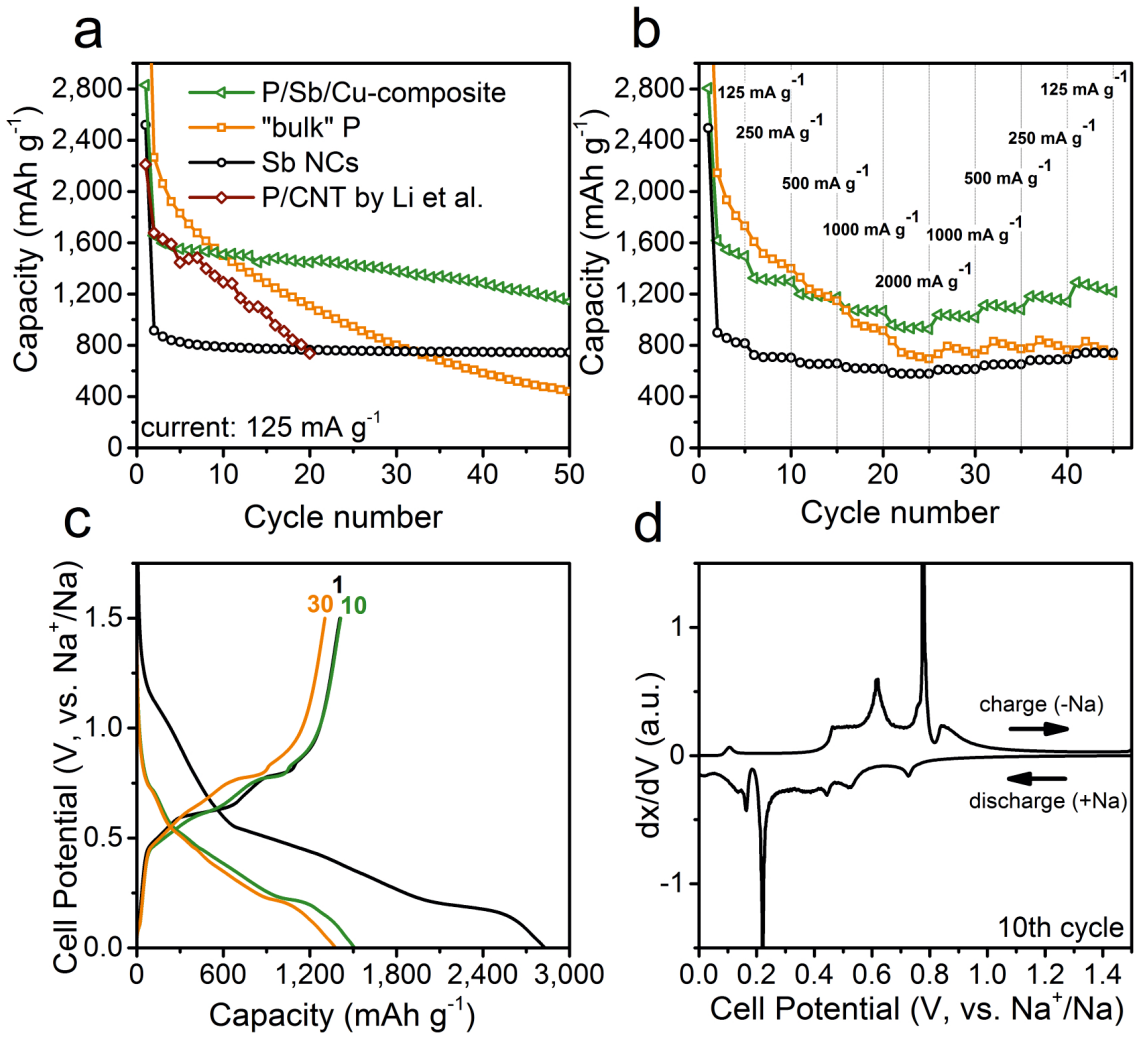
Electrochemical performance of the P/Sb/Cu composite electrodes in comparison to Sb NCs and red P: (a) cycling stability at a current density of 125 mA g^−1^; (b) rate-capability tests; (c) galvanostatic charge and discharge curves; (d) differential capacity plot for the 10^th^ cycle. The data of Li et al. for a hand-milled composite of red P and carbon nanotubes, tested under similar cycling conditions, are shown for comparison^24^. All electrodes were formulated as (active-material) :CB:CMC = 40%:40%:20% or, for samples containing Cu NWs, (active material) : CB : Cu : CMC = 40% : 30% : 10% : 20% by weight and cycled in the 0–1.5 V potential range.

**Figure 4:**
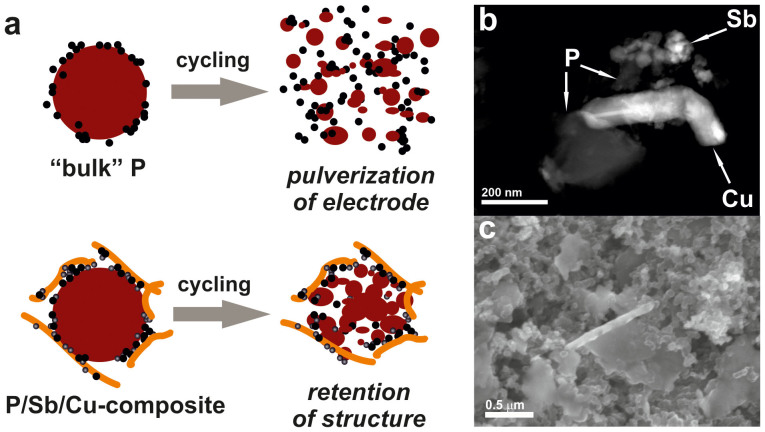
Schematics and characterization of the P/Sb/Cu composite: (a) Schematic depiction of the posited stabilizing effect of Cu NWs (with P in red, CB in black, Sb NCs in grey and Cu NWs in yellow); (b) HAADF-STEM image; (c) SEM image. The EDX-map corresponding to (b) can be found in the Supporting Information.

Figure 3 captures the main result of this communication – significant enhancement in reversibility of Na-ion storage by combining Sb NCs, “bulk” P and Cu NWs. Electrodes containing either Sb NCs, commercial red P or 1:1 mixtures thereof, with or without Cu NWs, were prepared with an overall mass ratio active-material:CB:CMC = 40%:40%:20%. Such relatively high fraction of conductive carbons such as CB are generally used for P-based electrodes in Na-ion batteries to provide sufficient electronic conductivity and mechanical stability of the electrodes^21^,^22^,^23^,^24^. Currents and capacities were related to the active mass without CB. From TEM and SEM images can be seen that the size of “bulk” P before and after mechanical mixing is retained (see Fig. 4 and Supplementary Fig. S4). As shown in Fig. 3, electrodes comprising 40% of pure Sb NCs deliver stable capacities and excellent rate capability. The fact, that at a current of 125 mA g^−1^ the apparent capacity of Sb NCs exceeds the theoretical value of 660 mAh g^−1^, can be explained by the contribution from CB (see Supplementary Fig. S5). At this current density, CB exhibits stable capacity of ca. 100 mAh g^−1^, and thus may be contributing ~100 mAh g^−1^ in its 1:1 mixture with Sb (or Sb/P). Electrodes composed of “bulk” P do show theoretical capacities in the first cycles, indicating that electronic connectivity is satisfactory in the as-prepared electrode, but undergo severe capacity fading to 440 mAh g^−1^ after 50 cycles. This result is fully consistent with the available literature on mechanically produced P/carbon electrodes^23^,^24^. For instance, Fig. 3a shows for comparison also the data of Li et al. for P/carbon nanotube (CNT)-composite, measured under similar conditions as in our work^24^. Experiments using 1:1 mixtures of “bulk” P and Sb NCs indicate electrochemical performance which exceeds the mathematic sum of the individual contributions, leading to retention of more than 800 mAh g^−1^ of capacity after 50 cycles (Supplementary Fig. S6). This improved capacity retention observed for electrodes of Sb/P electrodes can be explained by the lower content of P. Due to the higher „dilution“ of P particles in a matrix of Sb NCs and CB the volume changes can be buffered more effectively and electrodes are suffering from less mechanical stress leading to better cycling stability. Further major improvement has been attained by replacing some CB with Cu NWs. Cu NWs were synthesized according to a previously published procedure by Guo et al. (Supplementary Fig. S7)^58^. We note that Cu NWs are becoming comparably inexpensive. For instance, during the preparation of this manuscript, Li et al. reported facile synthesis of Cu NWs at a cost of 4.20 USD/g^59^. Further, it is generally well known that the cost of synthesis may further go down by up to an order of magnitude when material is produced on industrial scale. Electrodes comprising 1:1 mixtures of “bulk” P/Sb NCs with 10 wt% Cu NWs (i.e. 25% of CB was replaced with Cu NWs) delivered capacity of >1300 mAh g^−1^ for 30 cycles, corresponding to >80% of the theoretical capacity for this mixture (1628 mAh g^−1^). After 50 cycles still a capacity of >1100 mAh g^−1^ is retained, by 60% higher than in Cu-free samples and almost three times the value for “bulk” P, clearly manifesting the synergy between P and nanoscopic Sb and Cu.

An obvious benefit of the P/Sb/Cu formulation is seen also in the rate-capability tests (Fig. 3c). For comparison, all formulations of electrodes were subjected first to stepwise increase of the current density to 2000 mA g^−1^, and then to stepwise reduction of the current. Whereas electrodes of “bulk” P fail to recover their capacity, the P/Sb/Cu-composite is able to retain the same capacity level as after the continuous cycling at 125 mA g^−1^ (Fig. 3a). In addition, the P/Sb/Cu-composite shows excellent rate-capability with capacities of >900 mAh g^−1^ at 2000 mA g^−1^ current density.

An advantage of P is its lower electrode potential compared to Sb, potentially giving rise to higher energy density – that is higher voltage × capacity product of a full cell battery – when combined with high-voltage cathode. Figure 3c presents galvanostatic charge (desodiation) and discharge (sodiation) curves for P/Sb/Cu-composite, showing that sodiation and desodiation occurs at an average voltage of 0.5 V, highly suitable for anode applications. Additional hints can be found from differential capacitance plots (dQ/dV, Fig. 3d), derived from the charge/discharge curves, presented for the 10^th^ cycle (within the regime of stable cycling). In the first cycle (not shown here), two desodiation features at 0.6 and 0.8 V indicate individual contributions from P and Sb, as observed in reference electrodes comprising red P and Sb NCs (see Supplementary Fig. S8). However, in the subsequent cycles an additional broad component in 0.4–0.8 V range is built up, not present in Cu-free P/Sb electrodes. Further, we have observed that P/Sb electrodes exhibit an additional sodiation process at 0.16 V, not seen for electrodes composed of only P or Sb (see Supplementary Fig. S8). All other processes correspond to the individual contributions from P and Sb^23^,^52^. Temporal separation of electrochemical processes due to the spread of sodiation (desodiation) features over the broad voltage range may in fact have a stabilizing effect. In our recent study on Sn-Ge nanocomposites as LIB anodes^60^, as well as in numerous other reports on LIBs, stepwise lithiation and delithiation in composites of several electrode materials has been shown to enhance the mechanical stability, as compared to instant expansion of the whole electrode.

The coulombic efficiency – the ratio between the amounts of electrical charge spent for sodiation and desodiation processes in each cycle – provides an important insight into the reversibility of the charging/discharging processes. Due to the formation of the solid- electrolyte interface (SEI) the coulombic efficiency for the first cycle with P/Sb/Cu electrodes is only 60%, but reached 95% in the subsequent cycles at a current of 125 mA g^−1^. This initially low coulombic efficiency can be explained by the small size of Sb NCs and Cu NWs, which provides a large surface area for the irreversible electrolyte decomposition during the first discharge. During cycling at a relatively high current of 2000 mA g^−1^ the coulombic efficiency increased to 98%. As commonly reported for conversion anode materials with large volume work during cycling, the cracking of SEI layer leads to continuous decomposition of the electrolyte on the freshly exposed surfaces. This constant reformation of the SEI lowers the coulombic efficiency and long-term cycling stability due to degradation of both the electrodes and electrolytes.

## Discussion

Figure 4 depicts the stabilizing effect present in P/Sb/Cu electrodes. Cu NWs are assumed to cause better mechanic stability and improved electronic connectivity, slowing down the loss of electric contact due to pulverization of P particles. Cu NWs can trap fractured material to a degree that CB particles cannot provide. Overall, dilution of the (sub)micron-sized P particles, when mixed with Sb NCs and Cu NWs, most likely improves the overall mechanical stability of the electrode leading to retarded cracking and crumbling during cycling. High-angle angular dark field–scanning transmission electron images (HAADF-STEM, Fig. 4b), bright-field STEM images (Supplementary Fig. S9), elemental mapping by energy-dispersive X-ray spectrometry (EDX-map, Supplementary Fig. S9) and scanning electron microscopy images (SEM) clearly evidence intimate intermixing in P/Sb/Cu electrodes. We thus attribute the herein presented outstanding electrochemical properties of the P/Sb/Cu-composite to the effective embedding of “bulk” P in a Sb/Cu/CB matrix.

It is important to point out that of all measured electrodes only the ones comprising “bulk” P and Sb NCs with 10 wt% Cu NWs had clearly emerged as champion devices. Replacing Sb NCs with micrometre-sized commercial Sb led to significantly poorer performance (see Supplementary Fig. S10), very similar to purely P-based electrodes. Similarly, whereas electrodes containing both “bulk P” and Cu NWs exhibit higher cycling stability than in the absence of Cu NWs, they fall well behind the P/Sb/Cu-composite (see Supplementary Fig. S11). In addition, increasing the content of Cu NWs from 10 to 20 wt% did not improve the cycling stability further, but rather led to lower capacities (Supplementary Fig. S6). Considering that electrodes are composed of particles with very different size, this observation indicates that in order to obtain the optimal electrical contact in the electrodes and therefore highest capacity the ratio between CB particles and Cu NWs needs to be properly balanced. Finally, SEM images after extended galvanostatic cycling (Supplementary Fig. S12, 100 cycles) indicate that P particles become smaller, some cracks develop, but overall mechanical integrity of the electrodes is retained.

A great deal of work still has to be focused on improving the long-term cycling stability of the electrodes. Besides the effect of the active storage material, the cycling stability is a complex function of the electrode formulation (chemistry and amounts of binder and conductive additive), porosity, electrode thickness, electrolyte, temperature etc. In particular, coulombic efficiencies of ~92%, ~95%, and 97–98% for P, P/Sb/Cu and Sb electrodes respectively, indicate a continuous consumption of electrolyte for side anodic reactions - reformation of unstable SEI layer after each cycle and, very likely, reactivity of Na_3_P towards the electrolyte^23^. Smart engineering of the electrodes for instance by designing secondary structures combined with judicious choice of electrolytes and electrolyte additives will enable higher stability of SEI layer in future studies. We note that in the field of SIBs the problems of optimizing the chemistry of electrolytes and understanding of SEI formation had so far received much less attention as compared to 30 years of research on LIBs, and the knowledge are not necessarily interchangeable between these two fields.

In summary, this work showcases a strategy for constructing a composite Na-ion anode material, which combines several active materials with functional synergy between them. First, we presented a facile low-cost synthesis of Sb NCs that exhibit outstanding high-rate capability and long cycling life as anode material in SIBs. Compared to previous syntheses of Sb nanostructures by us and by others^53^,^61^,^62^,^63^, the advantages are the use of inexpensive reagents, the absence of any surfactants, simple washing procedure, high reaction yield and scalability. In fact, all that needs to be done for the synthesis of Sb NCs is addition of the Sb precursor solution at very moderate temperature (60°C) to the solution of NaBH_4_, which can easily be carried out on the industrial scale with either a batch or a continuous flow reactor. Purification of these Sb NCs involves only rinsing with water and filtering or centrifugation. A compelling P/Sb/Cu composite anode was then devised by simple mechanic mixing of Sb NCs with “bulk” red P and Cu NWs. In this composite, larger (sub)micron-sized P particles are embedded into a nanoscopic matrix of intimately intermixed Sb NCs, Cu NWs and nanoparticulate CB. Electrodes composed of 1:1 mixtures of “bulk” P/Sb NCs with 10 wt% Cu NWs delivered charge storage capacities of >1300 mAh g^−1^ for 30 cycles and >1100 mAh g^−1^ after 50 cycles at a current density of 125 mA g^−1^. Furthermore, reversible capacities of >900 mAh g^−1^ at high current density of 2000 mA g^−1^ have been obtained as well.

## Methods

### Synthesis of Sb NCs and Cu NWs

To synthesize ~20 nm Sb NCs, NaBH_4_ (48 mmol, 98%, ABCR) was dissolved in distilled N-Methyl-2-pyrrolidone (NMP, 51 mL, 99.8%, Fluorochem Ltd) in a three-necked flask under nitrogen and heated to 60°C. SbCl_3_ (12 mmol, 99%, ABCR) dissolved in NMP (9 mL) were quickly injected via syringe. The reaction mixture instantly turned black and was cooled down immediately using a water-ice bath. After cooling to room-temperature, Sb NCs have been separated from the solution by centrifugation (8000 rpm, 4 min) and washed three times with deionized water (30 mL) to remove unreacted NaBH_4_ and water-soluble side products such as NaCl. The reaction product was finally dried in the vacuum oven at room temperature, yielding 1.2 g of Sb NCs (82% reaction yield). NMP solvent recovered after centrifuging can be reused with or without distillation, giving very similar results. Cu NWs were synthesized according to a procedure published by Guo *et al*.^58^

### Electrode fabrication, cell assembly and electrochemical measurements

The following battery components were used: carbon black (Super C65, TIMCAL), carboxymethyl cellulose (CMC, Grade: 2200, Lot No. B1118282, Daicel Fine Chem Ltd.), NaClO_4_ (98%, Alfa Aesar, additionally dried), propylene carbonate (BASF, battery grade), 4-fluoro-1,3-dioxolan-2-one (FEC, Hisunny Chemical, battery grade), glass microfiber separator (GF/D, Cat No.1823-257, Whatman), and Cu foil (9 μm, MTI Corporation).

In a typical electrode preparation, the respective materials were combined with deionized water and mixed in a Fritsch Pulverisette 7 classic planetary mill for 1 h at 500 rpm. Mixing weight ratios were Sb:CB:CMC = 64%:21%:15% for pure Sb NCs (Fig. 2), and P/Sb:CB:CMC = 40%:40%:20% or P/Sb:CB:Cu:CMC = 40%:30%:10%:20% (Fig. 3). The aqueous slurries were coated onto Cu current collectors and then dried overnight at 80°C under vacuum prior to use. All electrochemical measurements were conducted in homemade, reusable and air-tight coin-type cells assembled in an Ar-filled glove box (O_2_ < 1 ppm, H_2_O < 1 ppm). Elemental sodium was employed as both reference and counter electrode. As electrolyte 1 M NaClO_4_ in propylene carbonate with 10% fluoroethylene carbonate was used. Glass fiber was used as separator. Galvanostatic cycling tests were carried out at room temperature on MPG2 multi-channel workstation (BioLogic). Capacities were normalized by the mass of active material.

### Materials characterization

Transmission Electron Microscopy (TEM) images were obtained with a Philips CM30 TEM microscope at 300 kV using carbon-coated Cu grids as substrates (Ted-Pella). Scanning transmission electron microscopy (STEM) and EDX mapping were performed on a JEOL 2200FS TEM/STEM microscope. Scanning electron microscopy (SEM) was performed using a NanoSEM 230. Powder X-ray diffraction (XRD) was measured on a STOE STADI P powder X-ray diffractometer.

## Supplementary Material

Supplementary Information Supplementary Information
