# Supplementary material for: Inexpensive Antimony Nanocrystals and Their Composites with Red Phosphorus as High-Performance Anode Materials for Na-ion Batteries
Source: Sci Rep. 2015 Feb 12;5:8418. doi: 10.1038/srep08418 (PMC4649624; doi:10.1038/srep08418)
Supplement: Supplementary Information [file srep08418-s1.pdf]

# Supplementary Information for

# Inexpensive Antimony Nanocrystals and Their Composites with Red Phosphorus as High-Performance Anode Materials for Na-ion Batteries

*Marc Walter,* [1, 2] *Rolf Erni,* [3] *and Maksym V. Kovalenko* [*1,2]

[1] Laboratory of Inorganic Chemistry, Department of Chemistry and Applied Biosciences, ETH Zürich, CH-8093 Zürich, Switzerland

[2] Laboratory for Thin Films and Photovoltaics, Empa – Swiss Federal Laboratories for Materials Science and Technology, CH-8600 Dübendorf, Switzerland

[3] Electron Microscopy Center, Empa – Swiss Federal Laboratories for Materials Science and Technology, CH-8600 Dübendorf, Switzerland

[*]E-mail: mvkovalenko@ethz.ch

**Table S1.** Calculated material cost for the as-prepared Sb NCs per gram of Sb NCs (1 CHF is approximately 1.1 USD). The cost of deionized water used for washing is negligible small and therefore not included.

| *Chemical* | *Amount used per 1 g Sb NCs* | *Relative Cost* | *Cost per 1 g Sb NCs* |
|---|---|---|---|
| NMP | 50 mL | 15.98 CHF/L | 0.80 CHF |
| $SbCl_3$ | 2.28 g (10 mmol) | 0.131 CHF/g | 0.30 CHF |
| $NaBH_4$ | 1.51 g (40 mmol) | 0.207 CHF/g | 0.31 CHF |
| | | | **Total: 1.41 CHF (1.55 USD)** |

**Table S2.** Comparison of the electrochemical performance of the herein presented P/Sb/Cu-composite with previously reported results obtained with P-based materials for Na-ion batteries.

| *Material* | *Preparation of composite* | *Initial capacity* | *Retained capacity* | *Cycle number* | *Reference* |
|---|---|---|---|---|---|
| P/Sb/Cu | mixing in aq. slurry (1h) | 1657 $mAhg^{-1}$ | 1376 $mAhg^{-1}$ | 30 | Present work |
| P/CNT | hand-grinding (1h) | ~1670 $mAhg^{-1}$ | ~730 $mAhg^{-1}$ | 20 | *Nano Lett.* **13**, 5480-5484 (2013) |
| P/C | ball-milling (20h) | ~1900 $mAhg^{-1}$ | ~1800 $mAhg^{-1}$ | 29 | *Adv. Mater.* **25**, 3045-3049 (2013) |
| P/C | ball-milling (24h) | ~1400 $mAhg^{-1}$ | ~1300 $mAhg^{-1}$ | 30 | *Angew. Chem. Int. Ed.* **52**, 4633-4636 (2013) |

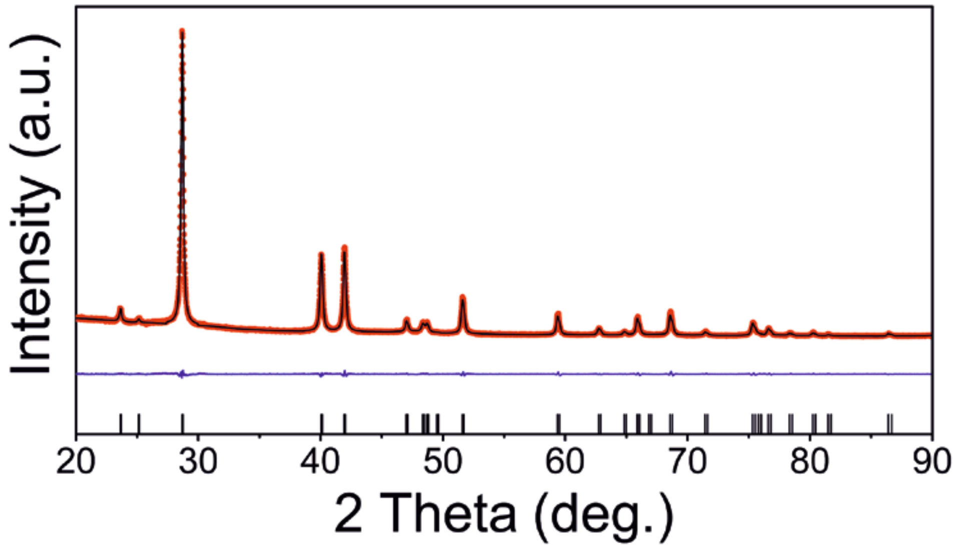


| Pattern | | | | Phase | |
|---|---|---|---|---|---|
| Chi2 | Rp | Rwp | Rexp | Rbragg | RF-factor |
| 2.396 | 12.9 | 11.3 | 7.29 | 0.954 | 1.81 |

**Figure S1.** Rietveld refinement (black curve) of the experimental XRD pattern (red curve) for Sb NCs. Blue line shows a difference curve. Refinement parameters are shown in a Table.

The crystallite size *D* was calculated from the formula:

$$D(\text{Å}) = \frac{180 \cdot K \cdot \lambda(\text{Å})}{\pi \cdot \sqrt{I_g}}$$

where $I_g$ is a parameter obtained by Rietveld refinement ($I_g$ = 0.156907), which accounts for the isotropic peak broadening due to the domain size, $K$ is the shape factor, $\lambda$ is the X-ray wavelength (Cu-K$\alpha$1 = 1.540598 Å). Rietveld refinement was carried out using FullProf Suite (https://www.ill.eu/sites/fullprof/).

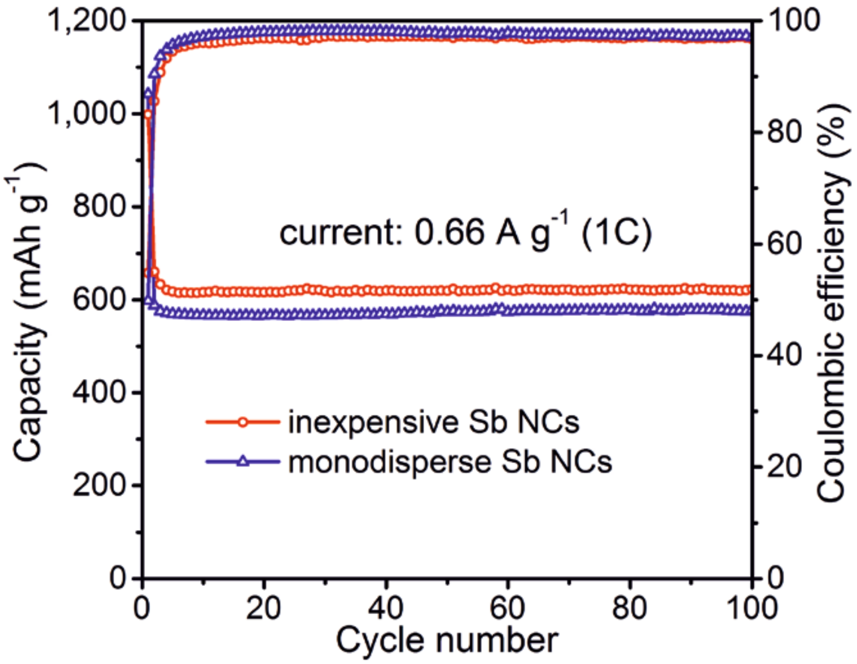


**Figure S2.** Comparison of the electrochemical performance of inexpensive Sb NCs developed in this study with organometalically-synthesized monodisperse Sb NCs from our previous report (He, M.; Kravchyk, K.; Walter, M.; Kovalenko, M. V. *Nano letters* **2014**, *14*, 1255). All cells were tested under identical conditions. Higher capacities of inexpensive Sb NCs (observed with statistical significance for 20 cells) can be attributed to their higher purity (no use of surfactants, all synthesis by-products are water-soluble and removed during washing).

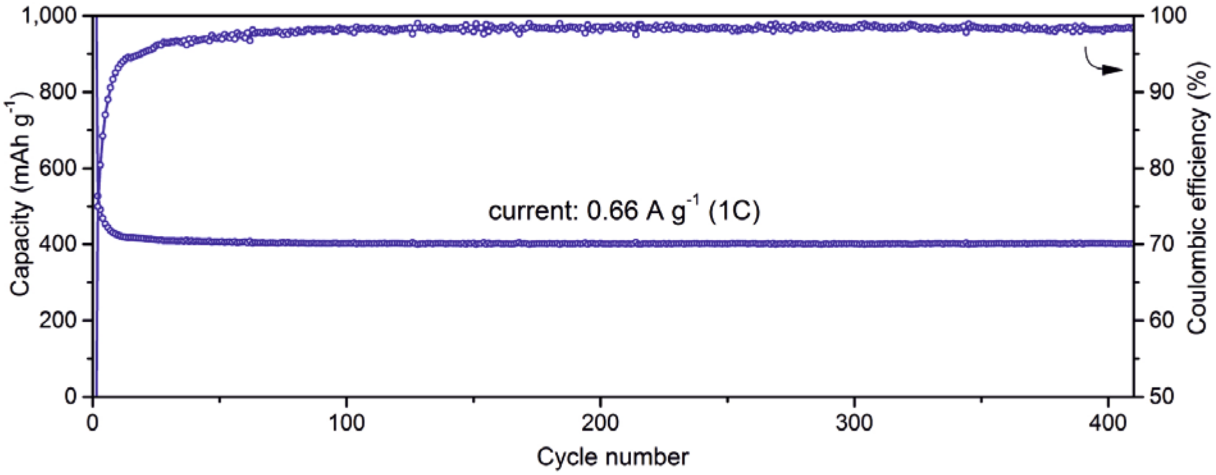


**Figure S3.** Long-term cycling stability of ~20nm Sb NCs in Na-ion coin-type half-cells with limitation of the charge capacity to 400 mAh g$^{-1}$.

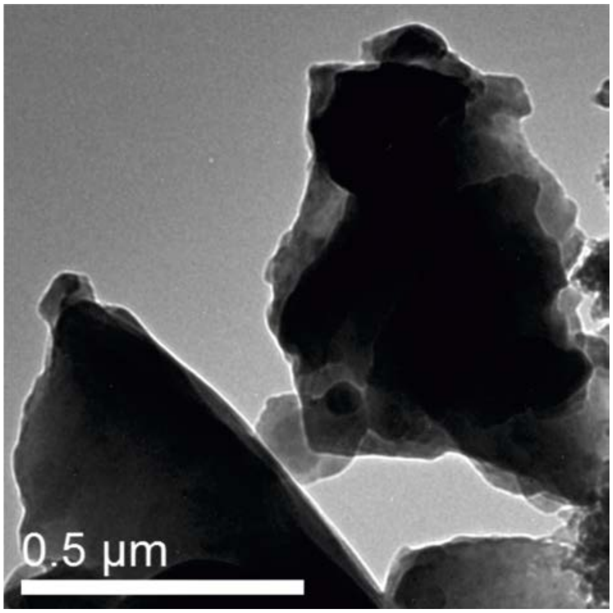


**Figure S4.** TEM-image of the as received commercial red phosphorus.

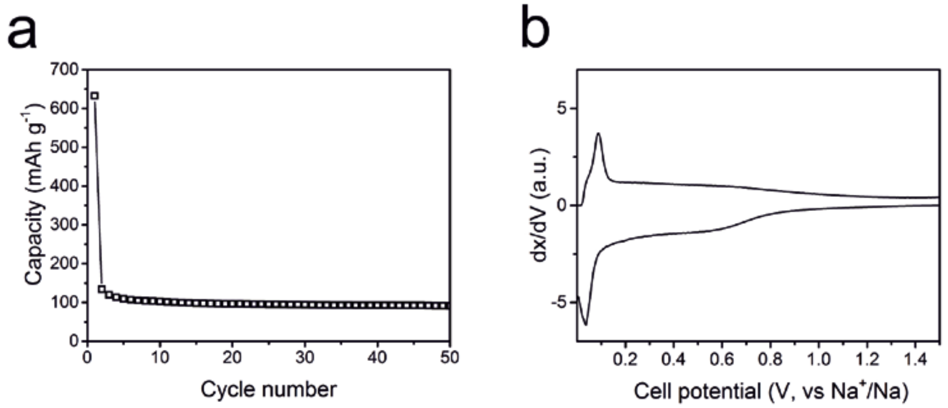


**Figure S5.** (**a**) Galvanostatic cycling of electrodes composed of 80% CB and 20% CMC at 125 mA $g^{-1}$ in the voltage range of 0 – 1.5 V; (**b**) Differential capacity plot for the 10$^{th}$ cycle.

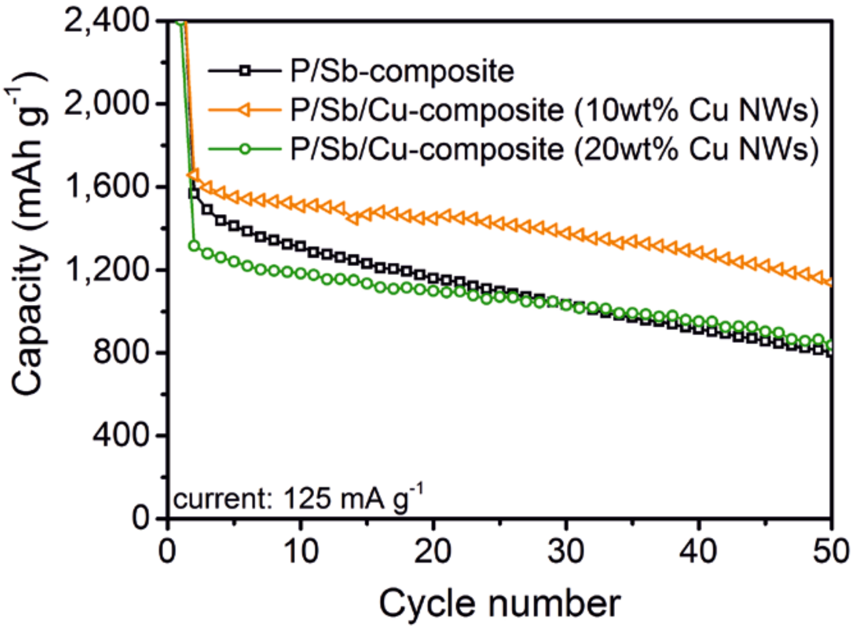


**Figure S6.** Galvanostatic cycling of electrodes composed of “bulk P” and Sb NCs (1:1) with and without 10% or 20% Cu NWs. Current: 125 mA $g^{-1}$; voltage range: 0 – 1.5 V; composition: 40% P/Sb (1:1) – 40% CB/Cu NWs – 20% CMC (by mass).

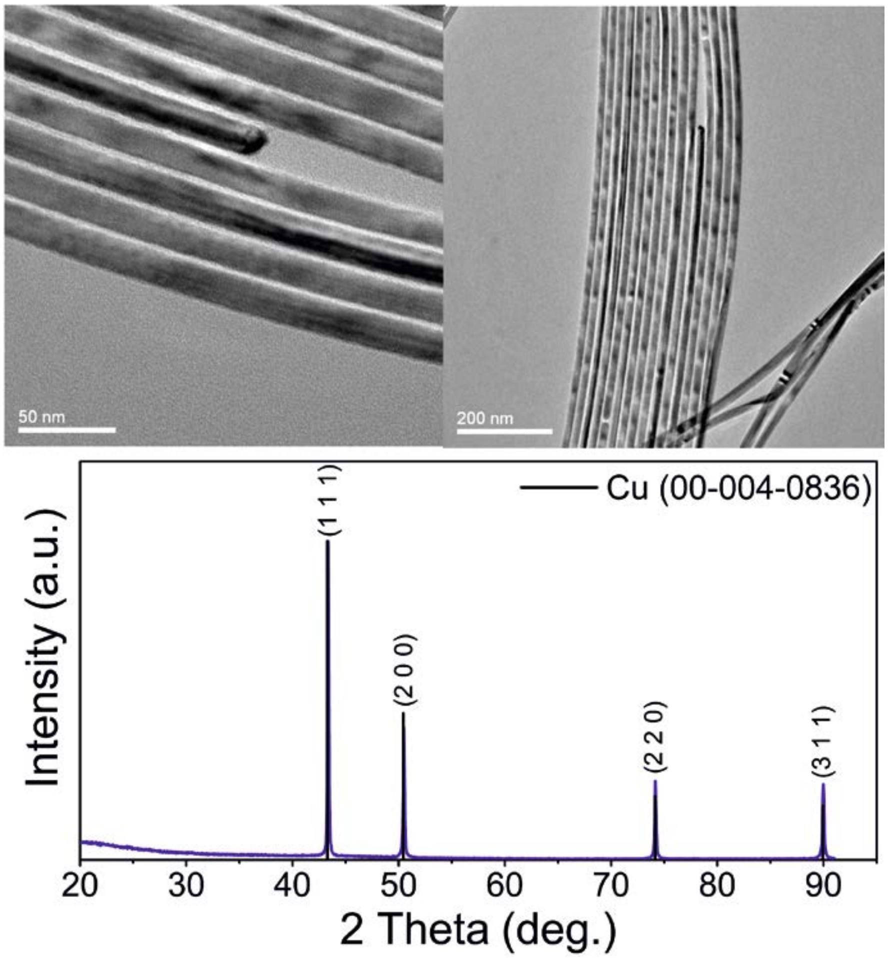


**Figure S7.** TEM images and XRD pattern of Cu NWs.

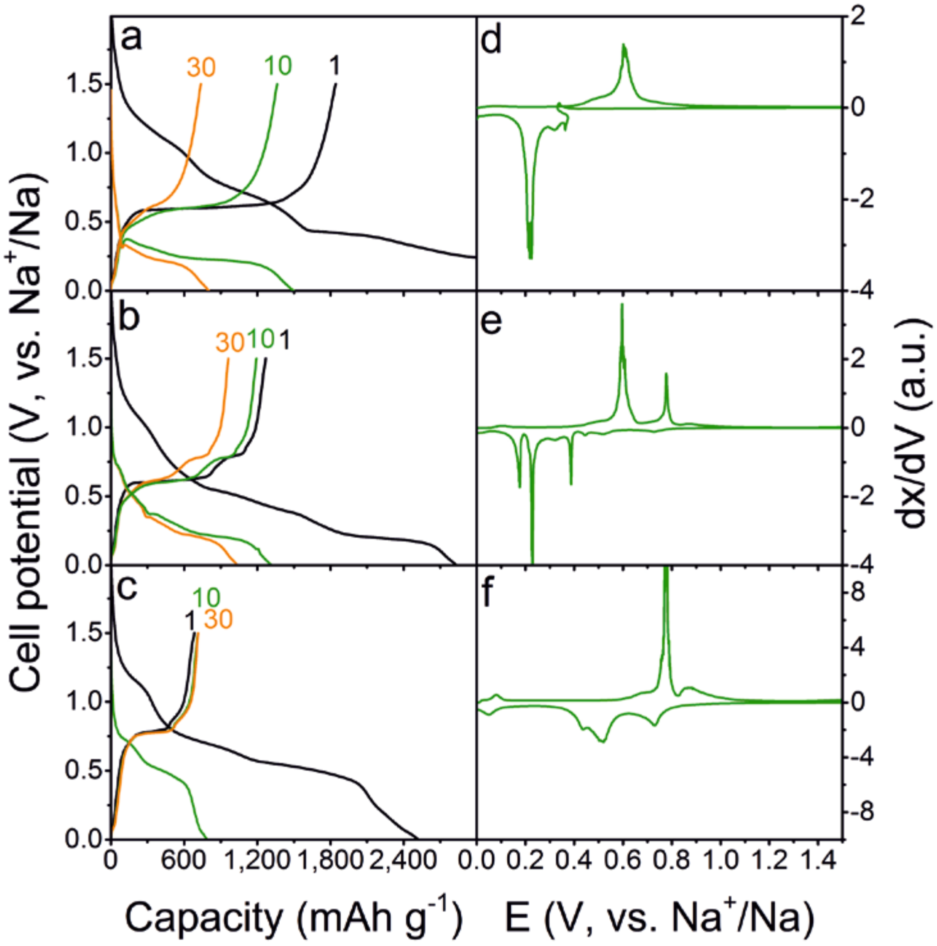


**Figure S8.** Galvanostatic charge/discharge curves for electrodes composed of "bulk" P (**a**), Sb NCs (**c**) and (**b**) 1:1 mixtures thereof; (**d-f**) differential capacity plots for the $10^{th}$ cycle. Current: 125 mA $g^{-1}$; potential range: 0 – 1.5 V; composition: 40% P/Sb – 40% CB – 20% CMC.

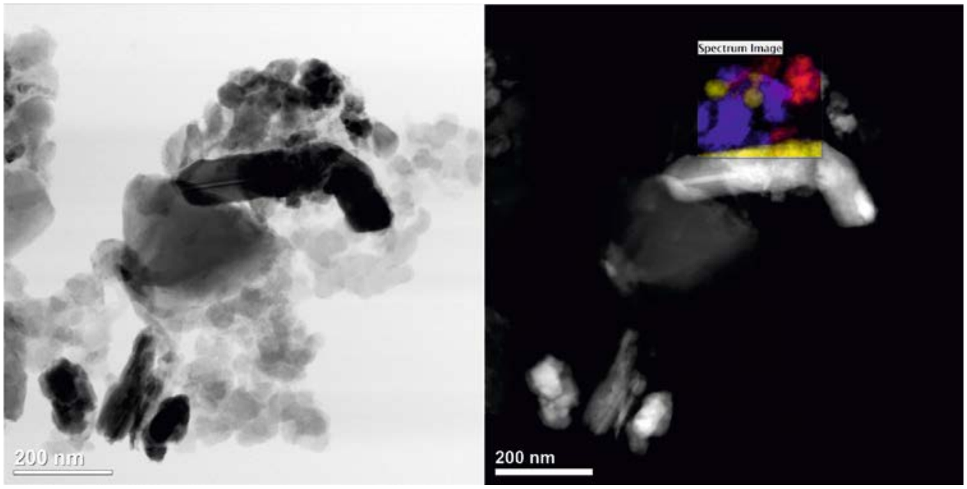


**Figure S9.** BF and HAADF-STEM images of the P/Sb/Cu-composite with an EDX-map as a colored inset (violet = P, red = Sb, yellow = Cu).

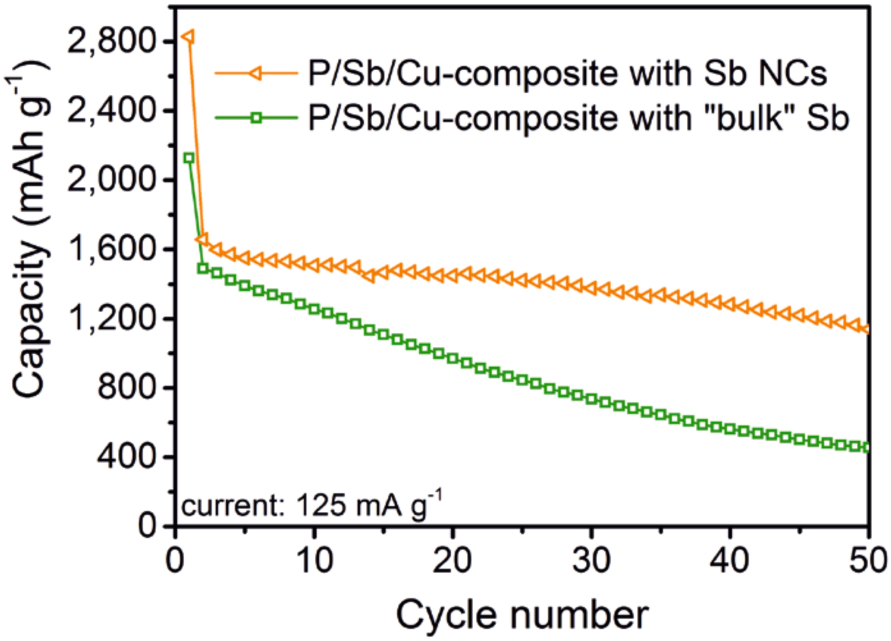


**Figure S10.** Galvanostatic cycling of P/Sb/Cu-composite prepared from Sb NCs or from microcrystalline Sb. Current: 125 mA $g^{-1}$; potential range: 0 – 1.5 V; composition: 40% P/Sb (1:1) – 30% CB – 10% Cu NWs – 20% CMC.

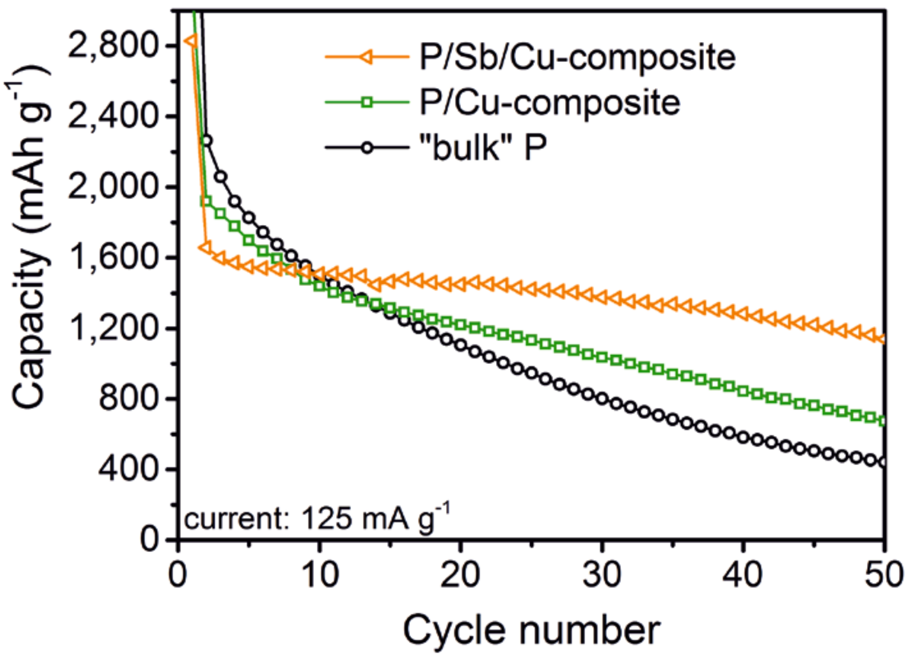


**Figure S11.** Galvanostatic cycling of electrodes composed of either "bulk" P, "bulk P" with 10wt% Cu NWs, or "bulk" P/Sb NCs (1:1) with 10wt% Cu NWs. Current: 125 mA $g^{-1}$; potential range: 0 – 1.5 V; composition: 40% P/Sb – 40% CB/Cu NWs – 20% CMC.

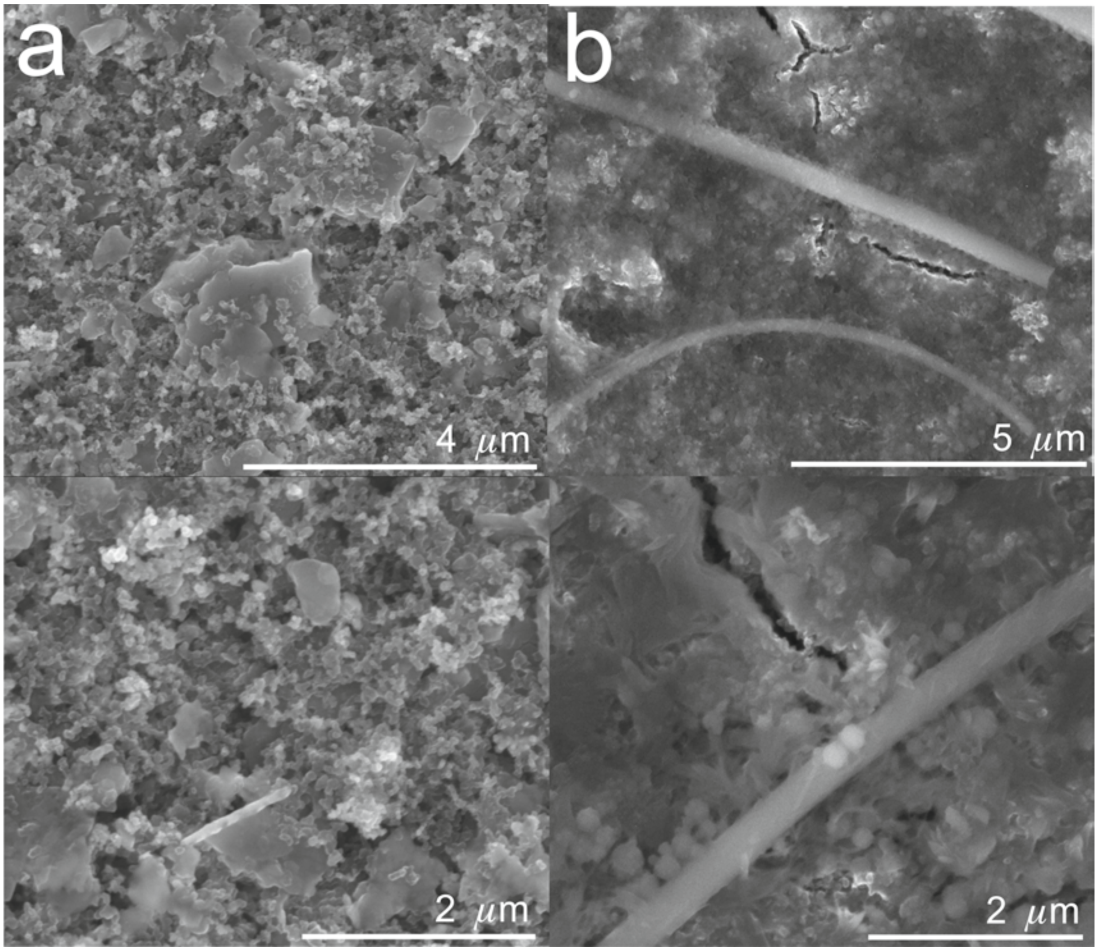


**Figure S12.** SEM images of electrodes composed of the P/Sb/Cu-composite before (**a**) and after (**b**) galvanostatic cycling experiments.

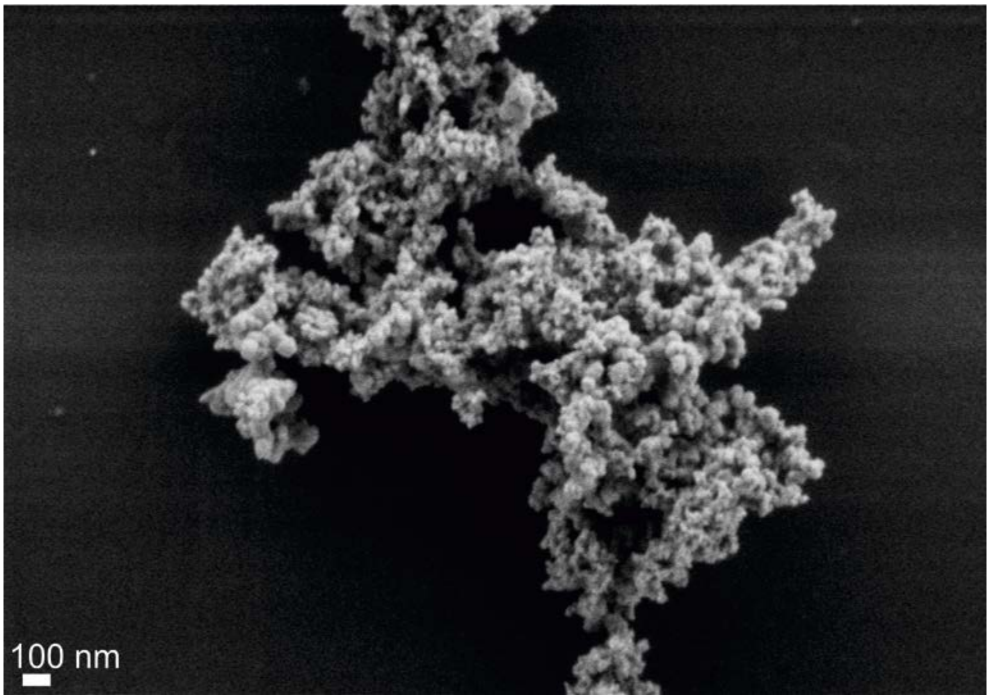


**Figure S13.** SEM image of the as-synthesized Sb NCs.
